# A Quantitative Study on the Trachea of the Red Sokoto (Maradi) Goat (*Capra hircus*)

**DOI:** 10.1155/2014/142715

**Published:** 2014-01-28

**Authors:** O. Byanet, J. A. Bosha, B. O. Onoja

**Affiliations:** ^1^Department of Veterinary Anatomy, College of Veterinary Medicine, University of Agriculture, Makurdi, Benue State, Nigeria; ^2^Department of Veterinary Physiology and Pharmacology, College of Veterinary Medicine, University of Agriculture, Makurdi, Benue State, Nigeria

## Abstract

The trachea forms the part of the conducting system which transports air from the external environment to the lungs. The aim of this study was to provide quantitative dimensions of the trachea of Red Sokoto goat (*Capra hircus*). Quantitative analysis was conducted on nine tracheas from goats (ages were ranged between eight months and three years) without sex variation in this study. The results showed that tracheas were extended from the cricoid cartilage of larynx to the hilus of the lungs, where they were divided into the right and left bronchi. They were structurally composed of the cartilaginous rings that were incomplete dorsally but bridged by tracheal muscles at the ends of the tracheal cartilages. The mean length of the trachea from the first to the last ring was 257 ± 7.11 mm and the number of tracheal rings varied from 35 to 57, with a mean value of 49.33 ± 2.78. The left bronchial mean length (19.78 ± 2.66 mm) was significantly longer than the right (10.44 ± 1.79 mm). The cross-sectional area (CSA) was wider at the intrathoracic area (221.5 ± 0.2 mm^2^) than cervical area (176 ± 0.1 mm^2^).

## 1. Introduction

Small ruminant sector plays an important role in the economic development of most West African countries. For example, in Nigeria, goats are kept by many rural dwellers in small herds to serve as sources of financial stability and supply of meat. In addition, some veterinary colleges/faculties use goats as a model for teaching gross anatomy practical class to undergraduate students. The most useful goat for these purposes is the Red Sokoto goat which is the predominant and most important breed of goat found mainly in the Sudan and Sahel savanna zone in the northwestern zone of Nigeria. This breed is well adapted to the arid zones and its population has been estimated to be 17.3 million, which accounts for about 70% of the 34.50 million goats in Nigeria [1].

Trachea is a heavy-walled tube extending from the cricoid cartilage of the larynx to the root of the lung where it bifurcates to form the right and left principal bronchia (*bronchus principalis*) [2]. The most distinctive features of the trachea are the cartilages (*cartilagines tracheales*) that form U-shape in animal like giraffe [3]. In the carnivores, these cartilaginous rings open in the dorsal wall of the trachea by a membranous structure (*paries membranaceus*) without cartilaginous support but bridge by tracheal muscles (*musculus trachaelis*) [4]. It has been documented that the tracheal muscles are attached to the perichondrium on the external surface of the rings [5]. In the dog and cat, this attachment is on the external surface of the cartilage, and in the sheep, the ends of the cartilages overlap with several plates of cartilage found between the ends of the tracheal cartilages just cranial to the bifurcation.

The number of tracheal cartilages is not constant in all species and even in the animals of the same species [5]. The ox has 50 tracheal rings with the average length of 65 cm, 4 cm width, and 5 cm height [6]. The horse has a longer length than the ox and higher number of rings (50–60), while the pig tracheal length ranges from 15 to 20 cm with 32–35 numbers of rings [2].

Tracheal morphometry is of importance because the dimensions of the trachea form the conducting portion of the respiratory tract and play a vital role in breathing [7]. Many factors are said to affect the shape and dimensions of the trachea in mammals. For example, tracheal diseases can cause alteration in the shape and compliance of the trachea and this affects the ability of the trachea to respond to change in the air flow and may result in complications such as tracheal collapse [5]. Other authors noted that diseases like hypoplasia [8] and collapsed tracheal rings [9, 10] in addition to trauma [11] can cause tracheal stenosis.

The anatomical structures of the trachea have been reported in the horse, camel, dog, ruminants [12–14], and giraffe, but no such a report could be cited in the literature with respect to Red Sokoto (Maradi) goat (*Capra hircus*). Therefore, the aim of this study was to report the quantitative dimensional features of the normal tracheal structure in the Red Sokoto goat. The results of these findings will be useful for teaching and research for undergraduate and postgraduate students of veterinary and animal science. In addition, it will provide surgeons and anaesthetists with valuable information about the tracheal length, cartilage numbers, and thickness of different regions in this breed.

## 2. Materials and Methods

### 2.1. Animal Source

Tracheas from nine goats (ages ranging from 8 months to 3 years) with no sex variations were used in this study. The goats were purchased from the animals market at Makurdi town of Benue State and transported by road to the Gross Anatomy Laboratory of the Department of Veterinary Anatomy, University of Agriculture, Makurdi, where they were embalmed and used in gross anatomy unit for teaching veterinary undergraduate students. There was no history or observed clinical signs related to the respiratory disease(s) in these animals.

### 2.2. Embalmment and Dissection

The embalmment protocol was done according to the described method [15]. Briefly, the carotid sheath was approached through the midventral incision in the neck region. The common carotid artery was dissected out on the right side and each animal was bled through this vessel by inserting an 18-gauge needle in the direction of the heart and securing it in position. Animals were then perfused with 8% formalin and 2% phenol in aqueous solution. The perfusion aspirator containing the formalin solution was kept at a height of 2.5 m from where the solution gravitated into one of the common carotid arteries through rubber tubing connected to a 6-gauge needle inserted into the artery. The nostrils were plugged with cotton wool before the fluid was let in and the perfusion was considered complete when fluid started leaking out through the nostrils and the anus. After two weeks of perfusion, fascia and muscles of the ventral aspect of the neck were gently dissected to expose the trachea. The whole trachea was then removed from the cricoid cartilage of the larynx to the lungs. The Nomina Anatomica Veterinaria [16] was used for nomenclature.

### 2.3. Morphometry

The entire length of the trachea was measured by twine and meter rule from the cranial border of the first tracheal ring to the bifurcation of tracheal rings as tracheal length (TL) and the number of the tracheal rings (TR) counted. Measurement from the first tracheal ring to the last ring before thoracic inlet was considered as the cervical region (CVR) and from that point to its bifurcation as intrathoracic region (ITR). The length from tracheal bifurcation to the hilus of the lungs was also measured as length of right bronchus (LR) and left bronchus (LB) (Table 1).

**Figure 1:**
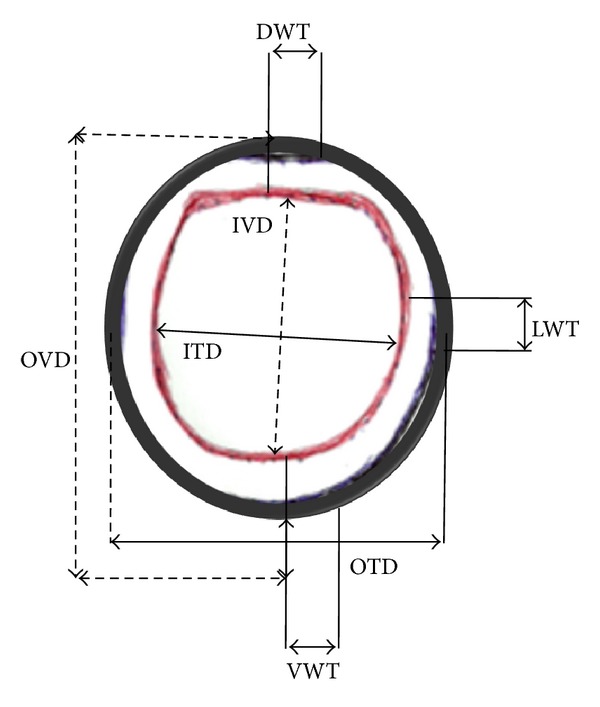
Schematic representation of the trachea showing where the inner transverse diameter (ITD), outer transverse diameter (OTD), inner vertical diameter (IVD), and outer vertical diameter (OVD) are. The thickness of the cartilaginous wall was measured on the dorsal, ventral, and lateral aspects as dorsal wall thickness (DWT), ventral wall thickness (VWT), and lateral wall thickness (LWT), respectively, were measured.

The tracheal annular ligament (*ligamenta anularia tracheae*) was incised and the outer vertical diameter (OVD), outer transverse diameter (OTD), the inner vertical diameter (IVD), and inner transverse diameter (ITD) were measured using digital vernier calipers (Table 2). Furthermore, by incising the same tracheal annular ligaments at the cranial cervical, caudal cervical, and intrathoracic tracheal regions, the outer vertical height (OVD) and inner vertical height (IVD), measurements were also taken (Table 2 and Figure 1). Furthermore, the thickness of the cartilaginous wall was measured on the dorsal, ventral, and lateral aspects as dorsal wall thickness (DWT), ventral wall thickness (VWT) and lateral wall thickness (LWT), respectively (Figure 1).

The cross-sectional lumen areas (CSA) were calculated using a mathematical equation (CSA = (IV/2 × IT/2) × 3.14), where IV is inner vertical and IT is inner transverse tracheal lumen as described by [13]. The inner and outer diameters (ID and OD), respectively, of various regions such as cervical (CVR) and intrathoracic region (ITR) were also measured and compared (Table 3).

### 2.4. Statistical Analysis

Data were tabulated and expressed as mean ± standard error of the mean (SEM). Student *t-*test and one-way ANOVA with Dunnett's posttest were performed using GraphPad Prism version 5.00 for Windows, GraphPad Software, San Diego, California, USA, http://www.graphpad.com/.  *P* value of ≤ 0.05 was considered significant.

## 3. Results

### 3.1. Gross Structures

The tracheal gross structure in this study was observed to be extended from the cricoid cartilage of the larynx to the tracheal bifurcation (*bifurcatio tracheae*), where it was divided into the right and left bronchi. It had the cervical part (*pars cervicalis*) and the thoracic part (*pars thoracica*). The cervical part dorsally was related to the esophagus and longus colli muscle, while on the lateral side it was related to the structures thyroid glands, carotid artery, jugular vein, and laryngeal nerves. Over the left atrium of the heart, the thoracic part was observed to be divided into right and left bronchi.

The components of the tracheal tube included structures like cartilaginous rings (*cartilagines tracheales*), the tracheal annular ligaments (*ligamenta anularia tracheae*) (which connect the cartilage rings), the muscular layer or trachealis muscles (*musculus trachealis*), and the mucous membrane. The rings were incomplete dorsally but bridged by tracheal muscles between the ends of the tracheal cartilages to be entirely membranous (*paries membranaceus*) without cartilaginous support. 

### 3.2. Morphometric Findings

The tracheal mean length from its first to the last ring was 257 ± 7.11 mm and the number of tracheal rings ranged from 35 to 57, with a mean value of 49.33 ± 2.78 (Table 1). The left bronchial mean length (19.78 ± 2.66 mm) was significantly longer than the right (10.44 ± 1.79 mm) (*P* < 0.01).

The mean OVD (16.06 ± 0.39 mm) was significantly higher than the mean OTD (14.02 ± 0.5 mm) (*P* < 0.05). In the same vein, the mean IVD (13.28 ± 0.41 mm) was significantly higher than the mean ITD (11.61 ± 0.41 mm) (*P* < 0.01) (Table 2). Furthermore, the mean cartilage wall thickness (2.0 ± 0.17 mm) observed on the ventral aspect, was slightly higher than those values noted on the dorsal (1.39 ± 0.29 mm) and lateral (1.61 ± 0.22 mm) aspects but was not statistically significant (*P* > 0.05) (Table 2).

In Table 3, the dimension of tracheal regions showed the outer diameter to have a significant higher mean value (16 ± 0.44 mm) against the value (14.05 ± 0.43 mm) for the inner diameter (*P* < 0.05). For intrathoracic region, the mean outer diameter (17.39 ± 0.58 mm) was slightly higher than the mean inner diameter (16.28 ± 0.32 mm) but was not statistically significant (*P* > 0.05). The value of CSA at ITR (221.5 ± 0.2 mm^2^) was higher than at CVR (176 ± 0.1 mm^2^) (Table 3).

## 4. Discussion

As observed by this study, the high mean tracheal length is very important for conducting airways and hence plays a fundamental role in breathing. Therefore, tracheal measurements are of anatomical importance. The trachea location, its tube-like nature and the muscles that connect the cartilages together in this study are not a new thing it has been well documented for domestic animals and man [3, 4, 13, 17].

In the present study, the mean tracheal length of 257 ± 7.11 mm for Red Sokoto breed of goat was slightly higher than the mean value of 25 cm earlier reported for other breeds of sheep [6]. Furthermore, our value was much higher than the 19.5 ± 0.64 cm for dogs [13] and 15–20 cm for pigs, but less than 65 cm for oxen [6], 70–80 cm for horses, and 87 ± 0.83 cm for camels [18]. In another research, the biomechanics of the giraffe's larynx and trachea in the male had mean tracheal length of 2.3 m [19]. The sexual dimorphism with respect to the length of trachea, which was not part of our study, has also been documented in man. For example, the average tracheal length was observed to be 12 cm in males and 10 cm in females (range 8–13 cm) [20, 21]. In a different report for man, it was showed that the length of trachea in an adult male was 11 cm and 10 cm in female and that the trachea increases when head is thrown back and turned to the side and with inspiration but decreases on expiration [22].

As the length of trachea varies, the number of the tracheal cartilage rings is also not constant in all species but varies even in animals of the same species [23]. This variation in numbers of tracheal rings between specimens is due to individual anatomical variations [24]. In this study, the range of 35–57 was observed, which was slightly higher than the 36–45 documented for dog [13] in one study and the 40–45 for the same dog in another study [6]. Higher values are seen in young Arabian camels (*Camelus dromedarius*) which have a range of 75–81 with a mean value of 77.1 ± 0.45 [18]. The numbers of tracheal cartilages do not change with the age and there are usually 15–20, but up to 26 have been reported [24].

The fusions of tracheal rings have been observed to be a common feature in man and some mammals [6]. A study of tracheas from 19 mix breeds of dogs showed that fusion in a large number of tracheal rings occurs mostly in the cranial cervical region (50%) and at the level of the thoracic inlet (30%) [13]. In the sheep, it was also reported that the ends of the tracheal rings overlap in the cranial portion of the trachea [6]. According to researchers, fusion at the cervical region is very important because of neck movement, especially a high degree of motion within the sagittal plane form by atlas and axial joint and axial-occipital joint [25]. In our study, there was no observed fusion of the tracheal rings in any tracheal region as reported in other mammals. A report related to our findings showed that tracheal rings fusion is mostly seen in pigs and least often in the ruminants [3].

## 5. Conclusions

The trachea in Red Sokoto breed of goat tends to be circular in shape, but with a gap at dorsal wall between the ends of each tracheal cartilage which results in that part being membranous (*paries membranaceus)*, that is, without cartilaginous support. Fusion of the cartilage rings was not observed in any region of the trachea. In this study, the trachea seems to be longer and the number of tracheal rings is higher than those reported for sheep, pigs, and dogs [6]. The result of this study is relevant to veterinary surgeons and anesthetists, for it provides valuable information on the tracheal length, cartilage numbers, and thickness in different regions in this breed.

## Figures and Tables

**Table 1:** Length of the trachea and number of trachea rings of Red Sokoto goat.

Parameter (mm)	Animal number (*n* = 9)									Mean ± SEM	*P* value
	1	2	3	4	5	6	7	8	9		
TL	270	230	275	290	272	245	242	259	230	257 ± 7.11	—
TR	35	38	53	56	56	55	50	57	44	49.33 ± 2.78	—
RB	12	6	7	15	4	19	16	5	10	10.44 ± 1.79	—
LB	15	25	15	19	14	19	17	15	39	19.78 ± 2.66	0.01

TL: tracheal length, TR: number of tracheal rings, RB: right bronchus length, LB: left bronchus length, *n*: sample size, and SEM: standard error of mean.

**Table 2:** The diameter and thickness of the trachea wall of the Red Sokoto goat.

Length (mm)	Animal number (*n* = 9)									Mean ± SEM	*P* value
	1	2	3	4	5	6	7	8	9		
OVD	17	16	16	17	15	17	14	17.5	15	16.06 ± 0.39	0.05
OTD	13.5	17	12.7	13	13	16	14	14	13	14.02 ± 0.5	

IVD	15	13	13.5	14	13	15	12	11.5	12.5	13.28 ± 0.41	0.01
ITD	10	13	11	11	11	14	12	11.5	11	11.61 ± 0.41	

DWT	1	2	1	3.5	1	1	1	1	1	1.39 ± 0.29	ns
VWT	2	1	2	2	3	2	2	2	2	2.0 ± 0.17	
LWT	1	1.5	3	2	2	1.5	1	1.5	1	1.61 ± 0.22	ns

OVD: outer vertical diameter, OTD: outer transverse diameter, IVD: inner vertical diameter, ITD: inner transverse diameter, DWT: dorsal wall thickness, VWT: ventral wall thickness, LWT: lateral wall thickness, CSR: cross-sectional area, *n*: sample size, SEM: standard error of mean, and ns: not significant.

**Table 3:** The diameter of the tracheal parts in the Red Sokoto goat.

Length (mm)	Animal number (*n* = 9)									Mean ± SEM	CSA
	1	2	3	4	5	6	7	8	9		
CVR											
ID	13	16	13.5	16	13	15	13	14	13	14.05 ± 0.4	176.6 ± 0.1
OD	15	17	15	18	15	18	15	15	16	16.0 ± 0.44*	

ITR											
ID	17	15	16	16.5	16	18	17	16	15	16.28 ± 0.28	221.5 ± 0.2
OD	13	17	18	18.5	18	18.5	18	17	18	17.39 ± 0.58	

CVR: cervical region, ITR: intrathoracic region, ID: inner diameter, OD: outer diameter, *n*: sample size, and SEM: standard error of mean; CSA: cross-sectional area; *P < 0.05.
